# Does mentalizing moderate the relationship between psychopathology and quality of life?

**DOI:** 10.1192/j.eurpsy.2023.1503

**Published:** 2023-07-19

**Authors:** B. Szabó, M. Miklósi, C. Sharp, J. Futó

**Affiliations:** 1 Department of Developmental and Clinical Child Psychology, Institute of Psychology, ELTE Eötvös Loránd University, Budapest, Hungary, Doctoral School of Psychology, ELTE Eötvös Loránd University, Budapest, Hungary; 2 Department of Developmental and Clinical Child Psychology, Institute of Psychology, ELTE Eötvös Loránd University, Budapest, Hungary, Institute of Psychology, ELTE Eötvös Loránd University, Budapest, Hungary; 3 Department of Psychology, University of Houston, Houston, United States

## Abstract

**Introduction:**

Most previous research focused on the association between mentalizing and specific mental disorders, while less is known about the relationship between mentalizing and quality of life among adolescents.

**Objectives:**

This study aimed to validate The Reflective Function Questionnaire for Youth in the Hungarian language and evaluate the moderating influence of mentalizing on the relationship between psychopathology and quality of life.

**Methods:**

A community sample of 384 youths of 12–18 years (72.7% females) completed the following questionnaires: The Reflective Function Questionnaire for Youth (RFQY), The Measure of Quality of Life for Children and Adolescents and The Strengths and Difficulties Questionnaire. First, we tested the different factor structures of the RFQY: the two-factor, the eight-item, and the five-item versions. We conducted a series of moderation analyses with quality of life as the dependent variable, higher-order symptom categories (internalizing or externalizing symptoms separately) as the independent variable, and mentalization as the moderator.

**Results:**

The confirmatory factor analysis supported the five-item version of the RFQY (Cronbach’s alpha .61) and resulted in a new, 10-item version of The Reflective Function Questionnaire for Youth on the Hungarian sample (Cronbach’s alpha .76). Mentalization had a moderator effect on the relationship between internalizing and externalizing symptoms and quality of life.

**Conclusions:**

Our study provides the first psychometric support for the Hungarian version of the RFQY and underlines the importance of assessing the complex relationships between mentalization, quality of life and symptomatology. Targeting mentalization to improve the quality of life among adolescents might be a key factor.

**Disclosure of Interest:**

None Declared

